# Hemicentin-1 is an essential extracellular matrix component of the dermal–epidermal and myotendinous junctions

**DOI:** 10.1038/s41598-021-96824-4

**Published:** 2021-09-09

**Authors:** Daniela Welcker, Cornelia Stein, Natalia Martins Feitosa, Joy Armistead, Jin-Li Zhang, Steffen Lütke, Andre Kleinridders, Jens C. Brüning, Sabine A. Eming, Gerhard Sengle, Anja Niehoff, Wilhelm Bloch, Matthias Hammerschmidt

**Affiliations:** 1 Institute of Zoology, Developmental Biology Unit, University of Cologne, Cologne, Germany; 2 Center for Molecular Medicine Cologne (CMMC), University of Cologne, Cologne, Germany; 3 Center for Biochemistry, University of Cologne, Cologne, Germany; 4 Department of Pediatrics and Adolescent Medicine, Faculty of Medicine and University Hospital Cologne, University of Cologne, Cologne, Germany; 5 Department Neuronal Control of Metabolism, Max-Planck Institute for Metabolism Research, Cologne, Germany; 6 Cologne Excellence Cluster on Cellular Stress Responses in Aging-Associated Diseases, University of Cologne, Cologne, Germany; 7 Department of Dermatology, Faculty of Medicine and University Hospital Cologne, University of Cologne, Cologne, Germany; 8 Cologne Center for Musculoskeletal Biomechanics (CCMB), University of Cologne, Cologne, Germany; 9 Institute of Biomechanics and Orthopaedics, German Sport University Cologne, Cologne, Germany; 10 Institute of Cardiology and Sports Medicine, German Sport University Cologne, Cologne, Germany; 11 Present Address: Instituto de Biodiversidade e Sustentabilidade—NUPEM/UFRJ, Universidade Federal Do Rio de Janeiro, Macaé, RJ Brazil; 12 Present Address: Molecular and Experimental Nutritional Medicine, Institute for Nutritional Sciences, University of Potsdam, Potsdam, Germany

**Keywords:** Biochemistry, Cell biology, Developmental biology

## Abstract

The extracellular matrix architecture is composed of supramolecular fibrillar networks that define tissue specific cellular microenvironments. Hemicentins (Hmcn1 and Hmcn2) are ancient and very large members (> 600 kDa) of the fibulin family, whose short members are known to guide proper morphology and functional behavior of specialized cell types predominantly in elastic tissues. However, the tissue distribution and function of Hemicentins within the cellular microenvironment of connective tissues has remained largely unknown. Performing in situ hybridization and immunofluorescence analyses, we found that mouse Hmcn1 and Hmcn2 show a complementary distribution throughout different tissues and developmental stages. In postnatal dermal–epidermal junctions (DEJ) and myotendinous junctions (MTJ), Hmcn1 is primarily produced by mesenchymal cells (fibroblasts, tenocytes), Hmcn2 by cells of epithelial origin (keratinocytes, myocytes). *Hmcn1*^−/−^ mice are viable and show no overt phenotypes in tissue tensile strength and locomotion tests. However, transmission electron microscopy revealed ultrastructural basement membrane (BM) alterations at the DEJ and MTJ of *Hmcn1*^−/−^ mice, pointing to a thus far unknown role of Hmcn1 for BM and connective tissue boundary integrity.

## Introduction

Extracellular microfibrillar networks composed of multi-domain extracellular matrix (ECM) proteins form intricate cellular microenvironments which are required to regulate tissue structure and function. The fibulin family of ECM proteins is of particular interest in this regard since they surround cells in close proximity to basement membranes (BMs) and the elastic fiber network, thereby guiding proper morphology and functional behavior of specialized cell types^1–4^. Hemicentins (Hmcn1, also named Fibulin-6, and Hmcn2) are ancient and very large members (> 600 kDa) of the fibulin family, however, their potential function within the cellular microenvironment of connective tissues remains largely unknown. Hmcns share a number of structural motifs. The most highly conserved is an amino-terminal von Willebrand A (VWA) domain, followed by a long (> 40) stretch of tandem immunoglobulin (Ig) domains, multiple tandem epidermal growth factor (EGF) domains and a fibulin carboxy-terminal module (FC). The carboxy-terminal EGF and FC modules combine to form a functional unit that is only found in Hemicentin-1, Hemicentin-2 and the other six members of the fibulin family (fibulin1-7)^5^.

Most functional investigations have been carried out in the nematode *C. elegans* where only one *hemicentin (hmcn)* gene is known, also referred to as *him-4*^6–8^. These studies point to pleiotropic roles of *hmcn* in transient cell contacts that are required for cell migration and basement membrane invasion, as well as in stable contacts at hemidesmosome-mediated cell junctions and elastic fiber-like structures^6,7^. Other studies identified Hmcn as part of an adhesion complex that connects juxtaposed BMs to each other, thereby conferring tissue linkage and structural integrity^9^. More recent studies using planarians as a highly regenerative and long-lived organism, nicely demonstrated that *hmcn* made by muscle cells as the main source of ECM proteins is required for BM integrity. In the absence of Hmcn protein, neoblast stem cells and their descendants become localized outside their normal compartments, pointing to essential roles of Hmcn and BM integrity for tissue separation^10,11^. Interestingly, also in mice, investigations in developing muscle focusing on the satellite cell stem cell niche highlighted Hmcn2 as a potential factor for correct BM-localization and development^12^.

Previous zebrafish data from our laboratory suggest that Hmcns synergize with other ECM proteins to define the ECM structure and development of various tissues. We identified zebrafish *hmcn1* mutants, which show fin blistering phenotypes similar to those of mutants in *fras1*, *frem2* and *fibrillin2* (*fbn2*), orthologues of human connective tissue disease genes encoding other ECM proteins^13^. On the ultrastructural level, this blistering is caused by compromised linkage of the epidermal BM to the underlying fin dermis, pointing to an essential role of Hmcn1 protein made by fin keratinocytes for proper dermal–epidermal junction (DEJ) formation in the developing fins^13^. Synergistic enhancement with specific antisense morpholino oligonucleotides (MOs) further showed that neither the *hmcn1* nor the *fbn2* MO-mediated knock-down alone elicited a phenotype at low MO doses, however when combined, they generated fin blisters similar to *hmcn1* mutants. These data strongly suggest that *hmcn1* and *fbn2* functionally interact during zebrafish fin development in vivo to allow proper fin DEJ formation and tissue linkage^14^. Similarly, it could be shown that *hmcn2* and *fibulin1* (*fbln1*) made by somitic muscle cells, thus stemming from internal sources, are required in a functionally redundant manner for DEJ formation in the trunk of zebrafish embryos^14^. Accordingly, *hmcn2*/*fbln1* double knock-down fish display blistering in the trunk only when both gene products were fully inhibited by high MO doses. Furthermore, *hmcn2*/*fbln1* double knock-down fish displayed impaired migration of fin mesenchymal cells into the fin folds, pointing to a crucial role of hmcn2 and fbln1 to remodel the ECM in the interepidermal space of the fin fold as a prerequisite for fibroblast ingrowth.

Until now, comparably little data has been gained about the tissue localization and function of Hemicentins in mammals. Recently, it was shown that Hmcn1 forms fine extracellular tracks along the BM of some elastic tissue structures in hair, the lumen of lymphoid conduits, and the mesangial matrix of the kidney glomerulus^15^. However, the functions of these tracks remain obscure, especially since genetic ablation of *Hmcn1*, *Hmcn2*, as well as the double knock-out generated via CRISPR/Cas9 technology did not result in overt phenotypic changes in these tissues^15^. Furthermore, due to the lack of specific Hmcn2 antibodies the tissue localization of Hmcn2 in mice has not been determined^15^. It is therefore not clear whether Hemicentins may functionally compensate for each other due to their mutual presence in specialized tissue microenvironments.

In this study, employing comparative and double in situ hybridization and immunofluorescence analyses, we demonstrate that Hmcn1 and Hmcn2 display complementary expression and distribution patterns throughout different developmental stages and within different tissues and tissue borders, including the DEJs and the myotendinous junctions (MTJs). Hmcn1 is mainly produced by mesenchymal cells, while Hmcn2 appears to be expressed by cells of epithelial origin. Consistent with previous reports^15^, *Hmcn1*^−/−^ mice generated in this study via conventional gene recombination technology, although lacking detectable Hmcn1 protein, are viable and show no obvious morphological, mechanical or locomotion phenotypes. However, transmission electron microscopy revealed ultrastructural BM alterations at the DEJ and MTJ of *Hmcn1*^−/−^ mice, pointing to a thus far unknown essential role of mouse Hmcn1 for BM integrity as the prerequisite for proper tissue linkage.

## Results

### Hmcn1 and Hmcn2 display differential distributions throughout development

**Figure 1:**
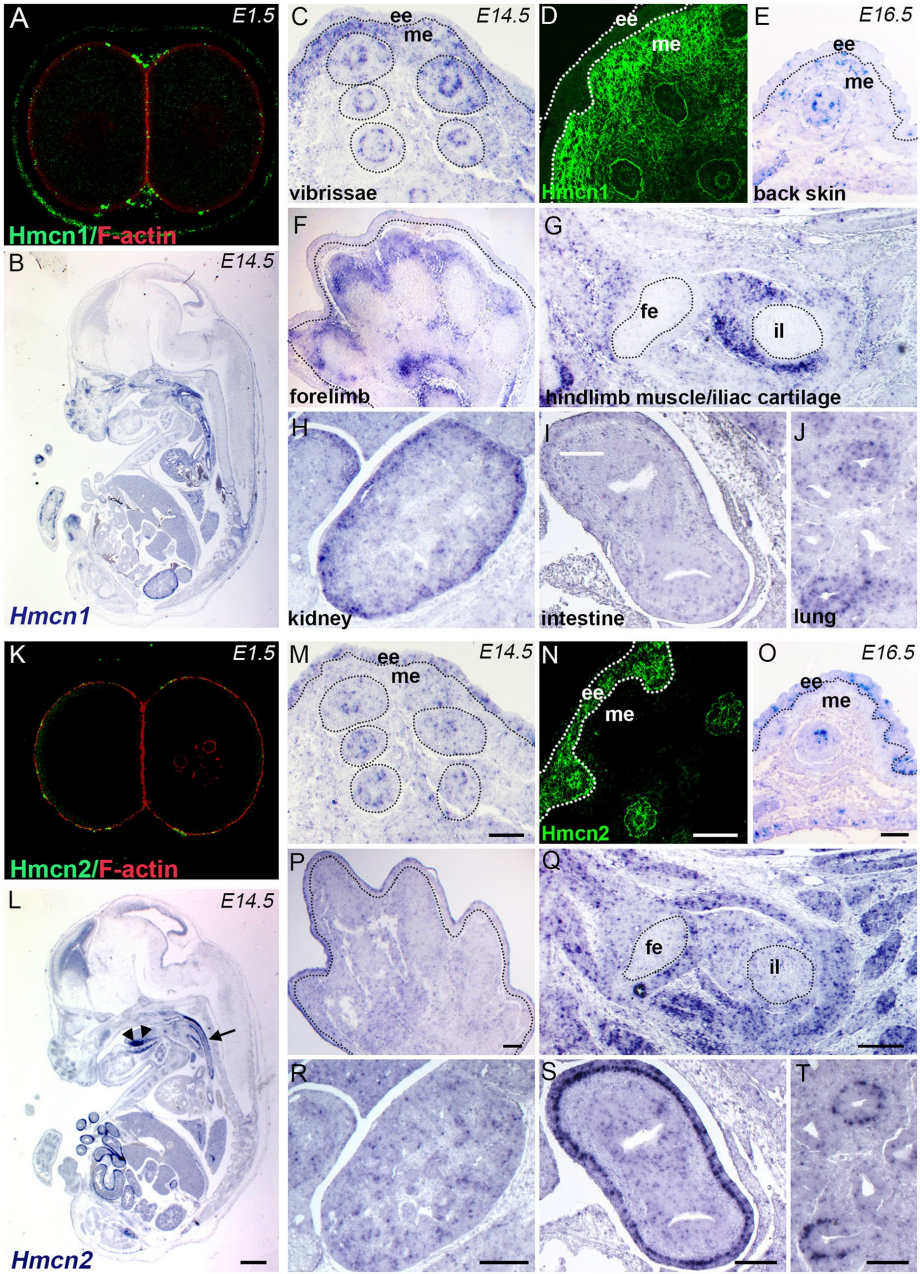
Differential expression of Hmcn1 and Hmcn2 in epithelial and mesenchymal cells of multiple tissues during murine embryonic development. Immunofluorescence images of two-cell stage (E1.5 days) of wild-type embryos using antibodies against mouse Hmcn1 (**A**, green) and Hmcn2 (**K**, green), counterstained with F-actin (red). (**B, L**) Sagittal section overview of an E14.5 embryo labelled by in situ hybridization with *Hmcn1* (**B**) and *Hmcn2* (**L**) probes. In (**L**) *Hmcn2* is strongly expressed in the tongue (arrowheads) and in the muscular layer of the esophagus (arrow). Unless indicated otherwise, following sections are at E14.5. (**C, M**) In vibrissae *Hmcn1*is expressed by mesenchymal cells (me), whereas *Hmcn2* is expressed by basal keratinocytes in embryonic epidermis (ee). (**D, N**) Immunofluorescence of Hmcn1 (**D**, green) confirming its localization in the extracellular matrix of the mesenchyme and Hmcn2 localization in the pericellular space of basal keratinocytes (**N**, green). (**E, O**) At E16.5, *Hmcn1* transcripts are present in the dermis, and *Hmcn2* transcripts in the epidermis of the back skin. (**F-J, P–T**) Differential expression of *Hmcn1* (**F-J**) and *Hmcn2* (**P–T**) in different embryonic tissues, as indicated. Scale bars **A**, **K** = 10 µm; **B**, **L** = 1 mm; **C**–**E**, **G**–**J**, **M**–**O**, **Q**–**T** = 100 µm; **F**, **P** = 200 µm. fe, femur; il, ilium.

In *C. elegans* and zebrafish, Hmcns have been shown to be essential for tissue morphogenesis and linkage^9,14^. Our previous results from mutagenesis screens in zebrafish implicated Hmcn1 in the pathology of Fraser syndrome which is characterized by developmental connective tissue defects such as soft tissue syndactyly (fusion of digits), and cryptophthalmos (fusion of the eye lids) as the result of embryonic skin blistering^13,16^. In mice, there are conflicting results about the importance of Hmcn1 for embryonic development. While it was initially reported that genetic ablation of *Hmcn1* in mice leads to preimplantation lethality^17^, a recent report showed that Hmcn1 is dispensable for murine embryogenesis^15^. To assess the functional roles of Hmcn1 and Hmcn2 during murine development, we analyzed their gene expression and their protein localization patterns at the two-cell stage (Fig. 1A, K) and at embryonic stages E14.5 (Fig. 1B–D, F–J, L–N, P–T) and E16.5 (Fig. 1E, O), performing in situ hybridizations (Fig. 1B, C, E–J, L, N, O–T) as well as immunofluorescence studies with Hmcn1- and Hmcn2-specific antibodies (Fig. 1A, D, K, N). Our immunofluorescence analysis showed that Hmcn1 localizes at the cleavage furrow during the two-cell stage (Fig. 1A), in line with previous reports using an antibody not discriminating between Hmcn1 and Hmcn2^17^. However, Hmcn2 is absent from the cleavage furrow (Fig. 1K). At E14.5, in situ hybridization of *Hmcn1* showed mesenchymal expression in the vibrissae, dermis, forelimbs, and kidneys (Fig. 1A, C–H). Also, *Hmcn1* probes yielded strong signals in intestine (Fig. 1I), lung (Fig. 1J), and in iliac cartilage (Fig. 1G). In these tissues, the two *Hmcn* genes appear to have a differential expression pattern with *Hmcn1* mostly expressed by mesenchymal cells, and *Hmcn2* primarily produced by epithelial cells. Similarly, immunofluorescence and in situ hybridization analysis revealed the presence of *Hmcn2* in the embryonic epidermis (Fig. 1M, N), while *Hmcn1* was exclusively present in the dermis (Fig. 1C, D). This complementary distribution in the two major compartments of the skin is still prevalent in later developmental stages such as E16.5 (Fig. 1E, O). This complementary expression suggests that Hmcns exert mutually exclusive functions during development*. Hmcn2* expression in kidney was negative, but in lung and intestine an epithelial *Hmcn2* expression was observed (Fig. 1R–T). Of note, *Hmcn2* is also expressed by muscular tissues such as skeletal hindlimb muscle (Fig. 1Q), tongue, and the muscular layers of the esophagus (Fig. 1L).

### Hmcn1 and Hmcn2 show complementary localization in postnatal connective tissues

**Figure 2:**
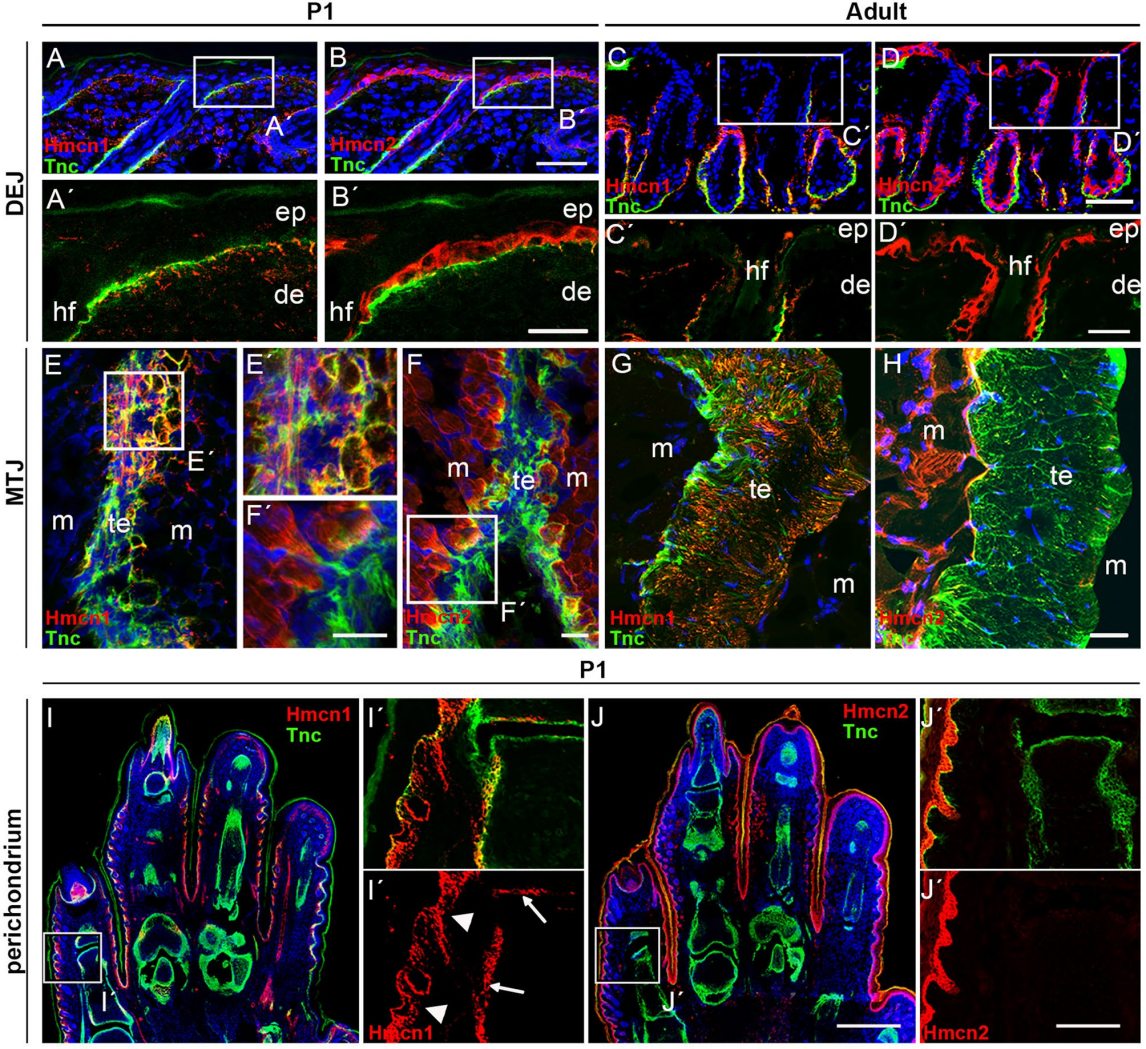
Complementary distribution of mouse Hmcn1 and Hmcn2 protein at the dermal–epidermal junction (DEJ), muscle–tendon junction (MTJ), and the perichondrium in neonates and adult mice. (**A**–**D**′) Immunofluorescent staining of DEJ of neonates/P1 (**A**–**B**′) and adult (**C**–**D**′) wild-type mice, counterstained for the ECM protein Tenascin C (green) and nuclear DNA (DAPI; blue). In neonates, Hmcn1 (red, **A, A**′) is localized throughout the dermis whereas Hmcn2 (red, **B, B**′) is distinctively localized in the pericellular space of basal keratinocytes of the epidermis. In adult, Hmcn1 is strongly concentrated on the dermal side of the basement membrane, but not detectable in the lower dermis (**C, C**′) and Hmcn2 is found in basal keratinocytes (**D, D**′). (**E–H**) Sagittal section of the calf in neonates (**E–F**′**)** and adult (**G, H**) mice, counterstained for the tendon ECM marker protein Tenascin C (green) and nuclear DNA (DAPI, blue). Hmcn1 (red, **E, E**′**, G**) is found within the tendon, forming long track-like structures and Hmcn2 (red, **F, F**′**, H**) in the endomysium of myofibers, with strong enrichment at the sites where myofibers are in close proximity to the tendon. (**I-J**′) Sagittal section of the autopod of newborn wild-type mice, counterstained with cartilage/bone ECM marker protein Tenascin C (green) and nuclear DNA (DAPI, blue). Hmcn1 (red, **I, I**′) is present in perichondrium (white arrows) and at dermal side of BM (arrowheads) Hmcn2 (red, **J, J**′) around basal keratinocytes of the epidermis. hf, hair follicle; ep, epidermis; de, dermis; m, muscle; te, tendon. Scale bars **A**–**D** = 50 µm; **A**′–**D**′, **E**–**H** = 25 µm; **I**, **J** = 400 µm; **I**′, **J**′ = 100 µm.

In postnatal murine skin, Hmcn1 and Hmcn2 maintain their compartment-specific localization established during developmental stages (Fig. 2A–D). In neonates, Hmcn1 is localized throughout the dermis (Fig. 2A, Fig. S2_1), whereas Hmcn2 is distinctively localized in the pericellular space of basal epidermal keratinocytes (Fig. 2B, Fig. S2_1). In adult skin, Hmcn1 is strongly concentrated at the dermal side of the basement membrane (BM), but not detectable in the deeper dermis (Fig. 2C), while Hmcn2 is restricted to basal keratinocytes of hair follicles and the interfollicular epidermis (Fig. 2D). To assess whether the observed compartment-dependent distribution of Hmcn proteins may change during skin regeneration, we analyzed full skin thickness excisional wounds at day 4 post injury by immunofluorescence. We found Hmcn1 to be up-regulated in the dermis adjacent to the epidermal tongues of closing wounds, while Hmcn2 appeared to be strongly up-regulated in the basal keratinocytes within the epidermal tongues (Fig. S2_2).

Hemicentins were also detected in postnatal musculoskeletal tissues, where they display a similarly complementary localization pattern. Immunofluorescence analysis of calf sections from neonates indicates the presence of Hmcn1 in tendons where it forms long track-like structures (Fig. 2E, G). Hmcn2 was found to localize within the endomysium of myofibers and appeared to be most enriched at the sites where myofibers are in close contact with tendons (Fig. 2F, H, Fig. S2_1). Overall, we observed a clear tendon-specific localization of Hmcn1 at the myotendinous junction (MTJ). In contrast, Hmcn2 is missing at the MTJ but clearly present in skeletal muscle itself (Fig. 2E–H, Fig. S2_1). Further, we detected Hmcn1 in the perichondrium of the murine autopod at P1 (Fig. 2I–I′), while in consecutive sections, Hmcn2 could only be found in the epidermis, but not in the perichondrium (Fig. 2J–J′). By employing immunohistochemistry, we could furthermore demonstrate Hmcn1 production by chondrocytes residing in articular cartilage and the femoral growth plate of 52 weeks old mice (Fig. S5_3).

### *Hmcn1*^−/−^ mice do not display any morphological phenotype during development

**Figure 3:**
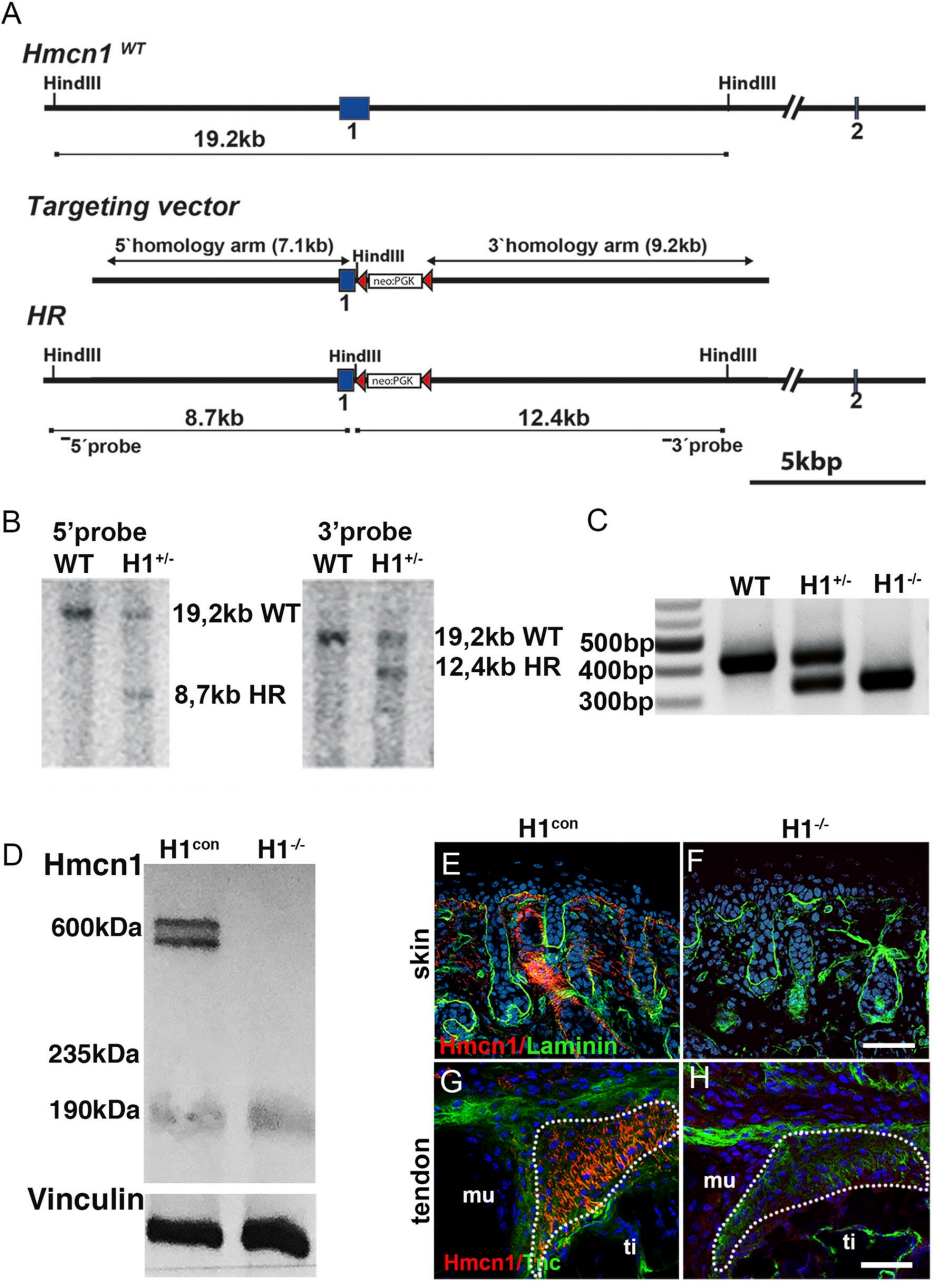
Generation of *Hmcn1* mutants by conventional gene targeting strategy. (**A**) Diagram representing wildtype locus of the *Hmcn1* gene (upper), the targeting vector used to generate the *Hmcn1* knock-out allele by partially substituting exon 1 by a neomycin resistance cassette (middle) and recombined DNA after homologous recombination (lower). The *frt* sites are shown as red triangles. The neo-cassette was removed after crossing chimeric mice with animals carrying the Flp-e deleter allele. (**B**) Verification of *Hmcn1* gene replacement by Southern blot analysis of *HindIII*-digested genomic DNA from ES cells using 5′and 3′probes demonstrated in A and by PCR analysis of mice using primers spanning exon 1 (**C**). (**D**) Western blot of skin lysates of adult mice showed the absence of Hmcn1 protein (bands at 600 kDa) in homozygous mutants. Vinculin probed on the same membrane is shown as a loading control. For full-size unprocessed images of (B-D), see Supplmental Information. (**E–H**) Confocal immunofluorescence of Hmcn1 (red) and laminin-α2 (green) in dermis (**E, F**) and tendon (**G, H**) of neonates indicates that Hmcn1 was not detected in H1^−/−^ mice (mu, muscle; ti, tibia). Scale bars represent 100 µm. H1^−/−^, *Hmcn1*^−/−^ mutants; H1^con^, sibling controls of *Hmcn1*^−/−^ mutants.

To investigate a functional role of Hmcn1 during development and postnatal homeostasis of connective tissues, we generated *Hmcn1* null mice by a conventional gene targeting strategy. Exon 1 of *Hmcn1* was partially substituted by introducing a neomycin cassette via homologous recombination (Fig. 3A). Knock-out of Hmcn1 was confirmed via Southern blot analysis (Fig. 3B) using 5′- and 3′-probes (indicated in Fig. 3A) and PCR using primers spanning exon 1 (Fig. 3C). Loss of Hmcn1 production was confirmed at the protein level via western blot and immunofluorescence analysis of skin and tendons of neonates and adults (Fig. 3D–H), using a Hmcn1-specific antibody raised against a fragment of the protein (1653–2275aa) containing several of the immunoglobulin (Ig) domains.

**Figure 4:**
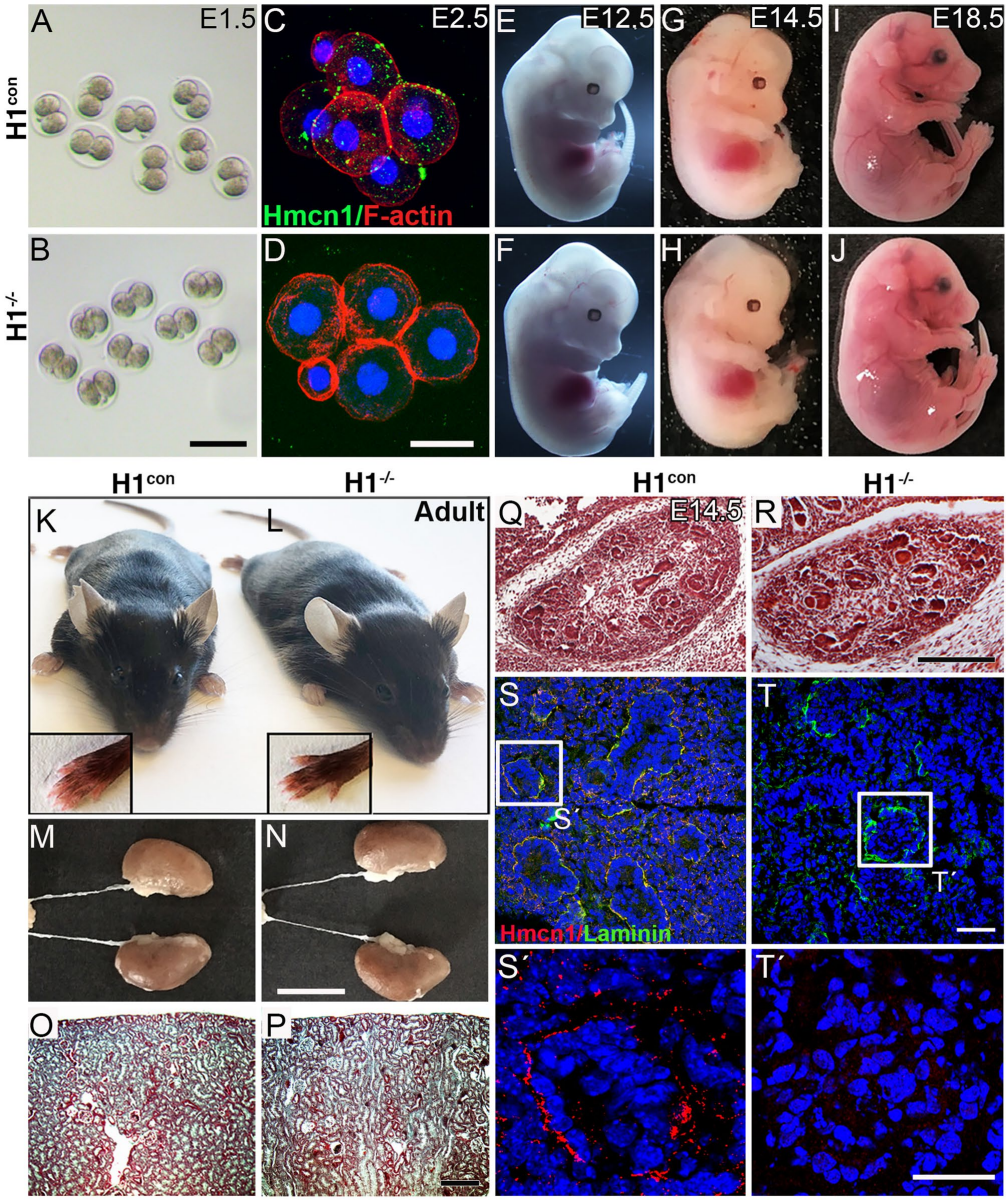
*Hmcn1* mutants show normal development, are viable, and lack previously described Fraser syndrome characteristics. Bright field image of 2-cell stage embryos (**A, B**) and immunofluorescence analysis of 6–8 cell stage H1^con^ and H1^−/−^ embryos (**C, D**). No detectable Hmcn1 protein in early development of H1^−/−^ embryos was observed. Different developmental stages of mouse embryos at E12.5 (**E, F**), E14.5 (**G, H**) and E18.5 (**I, J**) eliminate Hmcn1 gene as a potential Fraser syndrome candidate, as subepidermal haemorrhagic blisters were never observed in H1^−/−^ mice. Frontal view of control (**K**) and H1^−/−^ (**L**) adult mice. No cryptophthalmos (fusion of eyelids) or syndactyly (fusion of fingers/toes; insets) were seen. (**M, N**) Representative pictures of the urinary system with no renal hypoplasia or agenesis in H1^−/−^ adult mice. (**O, P**) Masson–Goldner Trichrome staining of cross-section of the adult kidneys did not reveal any differences between H1^con^ and H1^−/−^ mice. (**Q**–**T**′) Detailed analysis at E14.5 showed also no kidney phenotype in embryonic development as it was seen in *Fras1* mutants. Haematoxylin–eosin staining (**Q, R**) and immunofluorescence staining (**S, T**) with the indicated primary antibodies showed no difference in developing kidney in H1^−/−^ mice. In the wild-type control, Hmcn1 is localized at the BM of renal epithelia and in the ECM around nephron progenitor cells and of the renal stroma. (**S**′**, T**′) Higher magnification of the renal vesicle shows complete loss of Hmcn1 protein level in H1^−/−^ mice. Scale bars: **A**, **B** = 500 µm; **C**, **D**, **S**′, **T**′ = 25 µm; **M**, **N** = 1 cm; **O**–**R** = 200 µm; **S**, **T** = 50 µm. H1^−/−^, *Hmcn1*^−/−^ mutants; H1^con^, sibling controls of *Hmcn1*^−/−^ mutants.

Two previous studies reported conflicting data about the requirement of Hmcn1 for early murine embryogenesis^15,17^. Consistent with the most recent findings^15^, analysis of the two-cell stage and subsequent early developmental stages (e.g. E2.5) showed no morphological changes in *Hmcn1*^−/−^ mice, although they lacked detectable Hmcn1 protein (Fig. 4A–D). *Hmcn1* ablation also did not interfere with later embryogenesis, leading to normally developed mutant fetuses at expected Mendelian ratios (Fig. 4E–J). Furthermore, in contrast to our genetic studies in zebrafish, no Fraser syndrome-like symptoms such as subepidermal blistering, cryptophthalmos, or syndactyly were observed in adult *Hmcn1*^−/−^ mice (Fig. 4K, L). Also, histological analysis of fetal and adult kidneys did not reveal any morphological differences (Fig. 4M–R), another phenotypic trait of Fraser syndrome, although the mutants lacked Hmcn1 normally present at the BM of renal epithelia and in the ECM around nephron progenitor cells and in renal stroma (Fig. 4S, T).

### *Hmcn1*^*−/−*^ mice do not have any gross skin or musculoskeletal defects

**Figure 5:**
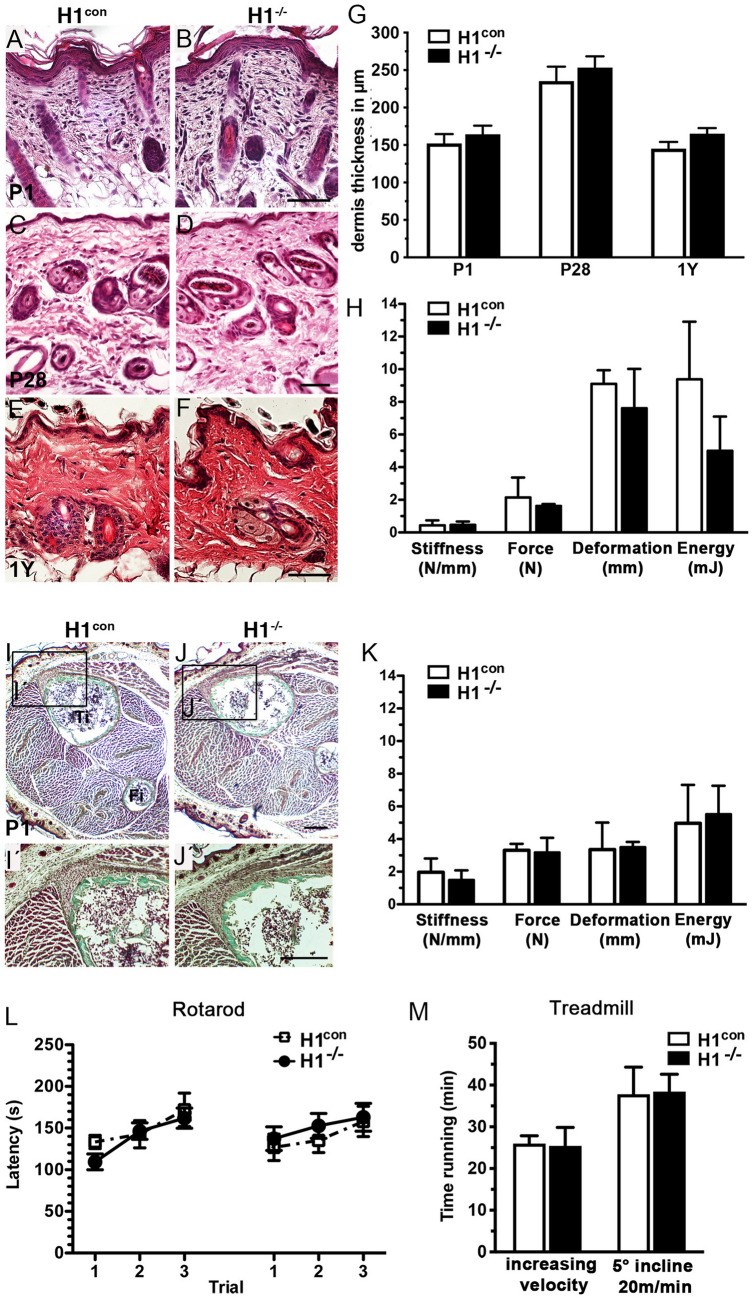
*Hmcn1* mutants have no gross skin or musculoskeletal defects. Morphological and histological analysis by hematoxylin–eosin staining of H1^con^ and H1^−/−^ in neonates (P1) (**A, B**), and in three weeks (P28) (**C, D**) and one-year old (1Y) (**E, F**) mice. (**G**) Dermis thickness was measured in µm, revealing no significant difference between of H1^−/−^ mutants and controls at any of the tested time points. (**H**) Back skin tensile strength measurements were performed on 16-months old male mice (5 H1^−/−^ mutants, 6 H1^con^ mice). After preloading specimen (0.05 N, 0.1 mm/s) the ultimate load (N), deformation (mm), energy (mJ) and stiffness (N/mm) were determined. (**I**, **J**) Masson–Goldner Trichrome staining on newly born H1^con^ and H1^−/−^ did not show any defects in muscle/tendon macroscopic structures. (**K**) Tendon/muscle strength measurements were performed on the same 16-months old male mice used for (H). After preloading specimen (0.01 N, 10 mm/min) the ultimate load (N), deformation (mm), energy (mJ) and stiffness (N/mm) were determined. (**L**) H1^con^ and H1^−/−^ mice adult males did not display any significantly different performance in rotarod tests. Latencies were measured in three consecutive trials with interjacent 15 min breaks (1, 2, 3) and on two consecutive days. (**M**) Measurements of maximum run time during the treadmill fatigue test revealed no significant changes between H1^con^ and H1^−/−^ mice. Two different tests were performed (i) with increasing velocity from 8 to 24 m/min for max 27 min (left side of the graph) and (ii) with constant speed of 20 m/min for 40 min and incline of + 5° (right side of the graph). Scale bars: **A**–**F** = 50 µm, **I**–**J**′ = 200 µm. Ti, tibia; Fi, fibula; H1^−/−^, *Hmcn1*^−/−^ mutants; H1^con^, sibling controls of *Hmcn1*^−/−^ mutants.

Since *Hmcn1* null mice did not show any major connective tissue defects during embryonic and fetal development, we investigated skin, muscle, and bone at different postnatal time points. No changes in skin morphology and thickness were observed in *Hmcn1*^−/−^ mice at P1, P28, and one year of age (Fig. 5A–G, Fig. S5_1). Additionally, muscle morphology remained unaltered in *Hmcn1*^−/−^ mice at all indicated time points (Fig. 5I–J).

*Hmcn1*^−/−^ mice had a normal weight at 14 months, and gait analysis as well as limb clasping assays at 6- and 14-months of age did not reveal any significant differences to wild-type controls (Fig. S5_2). Since Hmcn1 was detected in the microenvironment of femoral articular cartilage and hypertrophic growth plate chondrocytes, we also searched for potential detrimental consequences for femoral tissue structure caused by the loss of *Hmcn1*. However, µCT analysis of femurs showed no alterations in bone structural properties in *Hmcn1*^−/−^ mice (Fig. S5_3).

To determine whether Hmcn1 ablation leads to a change in biomechanical properties due to decreased connective tissue integrity, we challenged skin and skeletal muscle from 16 months old mice by mechanical loading experiments. However, tensile strength measurements of back skin and myotendinous junctions isolated from *Hmcn1*^−/−^ mice did not reveal any significant differences in biomechanical properties such as ultimate tensile strength (force), stiffness, or strain energy (Fig. 5H, K). Rotarod motor coordination and treadmill fatigue tests also did not indicate any significant changes of *Hmcn1* mutants compared to controls (Fig. 5L, M).

### *Hmcn1*^−/−^ mice display ultrastructural changes in basement membrane organization

**Figure 6:**
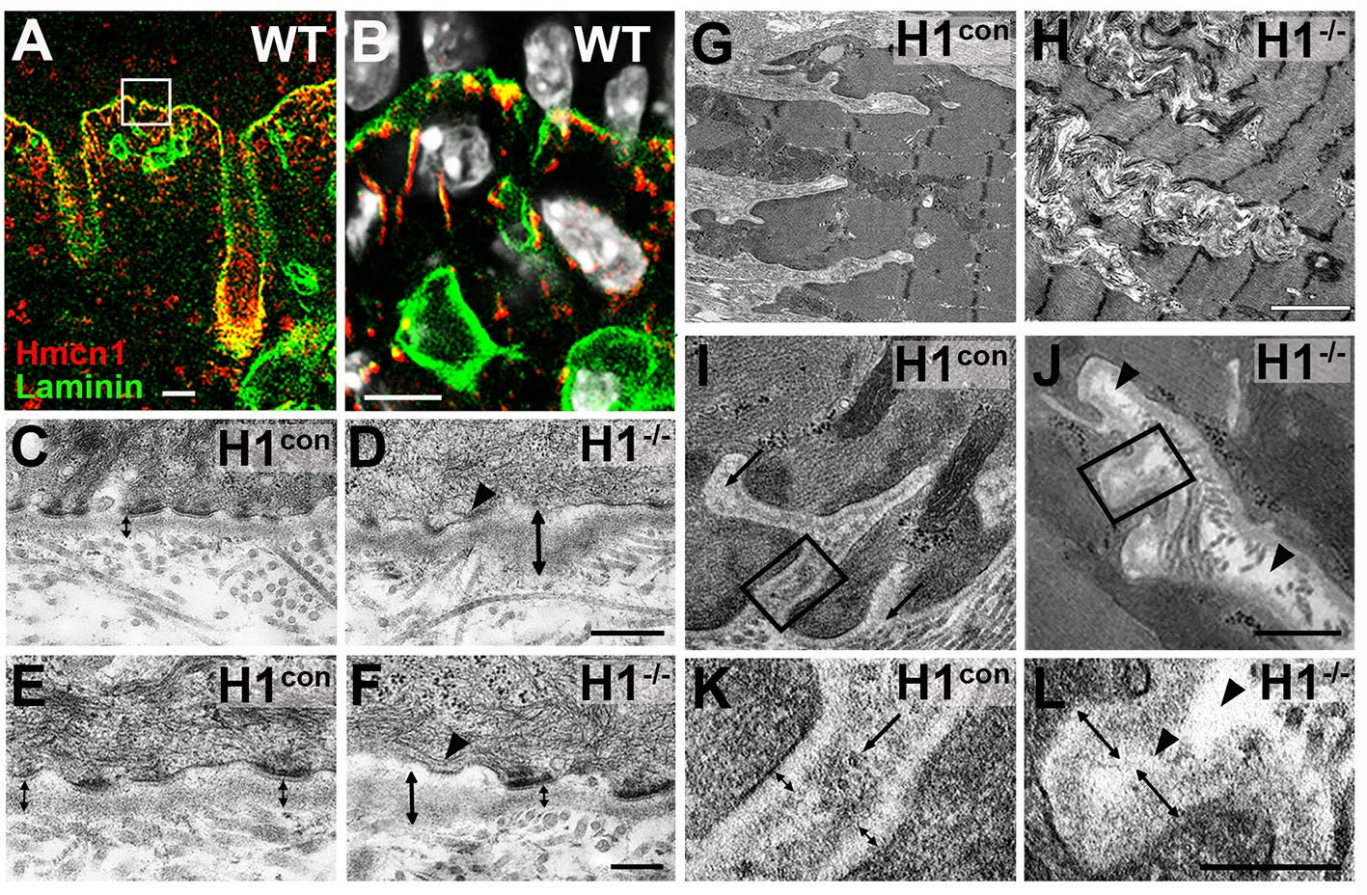
Ultrastructural changes in basement membranes and the organization of dermal-epidermal and myotendinous junctions of *Hmcn1* mutants. (**A, B**) Co-immunolabeling visualized by confocal immunofluorescence microscopy shows partial co-localization of Hmcn1 with the BM marker laminin-α2 at epidermal-dermal junction of back skin; B shows magnified view of region boxed in A; nuclear DNA stained with DAPI (white). (**C**–**F**) TEM analysis of murine back skin revealed a thickening of the BM in absence of Hmcn1 (**D, F**) compared to wild-type siblings (**C, E**). Bidirectional arrows mark area occupied by lamina densa and lamina lucida of dermal-epidermal BM, arrowheads in D, F point to hemidesmosomes of compromised morphology. (**G, H**) TEM analysis shows abnormal tendinous interdigitation patterns and ECM deposition at the myotendinous junctions of *Hmcn1*^−/−^ mice (G) compared to wild-type siblings (H). (**I–L**) Similar to identified BM alteration at DEJ, the BM at MTJ of *Hmcn1*^−/−^ mice is thickened. Bidirectional arrows mark area occupied by lamina densa and lamina lucida. The tips of the tendon interdigitations of wild-type controls harbor collagen fibers (arrows in **I**, **K**) adjacent to the MTJ BM, whereas the interdigitation tips of *Hmcn1*^−/−^ mice are largely devoid of collagen fibers (arrowheads in **J**, **L**). **K**, **L** show magnified views of regions boxed in (**I**, **J**). Scale bars: **A** = 10 µm; **B** = 5 µm; **C, D** = 250 nm; **E, F** = 150 nm; **G, H** = 1.5 µm; **I, J** = 500 nm; **K**,** L** = 250 nm. H1^−/−^, *Hmcn1*^−/−^ mutants; H1^con^, sibling controls of *Hmcn1*^−/−^ mutants.

Despite the apparent absence of gross anatomical, histological, mechanical and locomotion abnormalities in *Hmcn1* mutant mice, and in light of the described requirement of Hmcn in BM integrity and tissue linkage or separation in nematodes and flatworms (see “Introduction”), we next analyzed Hmcn1 mutants and in particular their BMs at the ultrastructural level. Like its invertebrate relative, mammalian Hmcn1 has been described as a BM-associated protein^9,15^. Indeed, performing co-immunofluorescence studies, we found Hmcn1 to co-localize with laminin-α2 at BMs around hair follicles in the skin/dermal-epidermal junctions (DEJs; Fig. 6A, B) as well as at myotendinous junctions (MTJs). To investigate in more detail potential BM defects at such DEJs and MTJs due to absence of Hmcn1, we employed transmission electron microscopy (TEM). Indeed, we observed moderate, but consistent ultrastructural alterations at both connective tissue junctions of adult *Hmcn1* mutants. In skin, the DEJ appeared irregular, with regions of wild-type morphology alternating with regions in which the BM showed an increased thickness due to a slightly widened lamina lucida and a distinctly widened and structurally altered lamina densa (Fig. 6C–F; n = 5/5 mutants). Analyzing the DEJ over a total length of approximately 2 mm from three independent mutants and wild-type siblings, regions with widened BM constituted 21.5 ± 6.9% of the entire DEJ of the mutants, compared to 2.1 ± 0.5% in the controls (*p* = 0.05). Also, in regions with increased BM thickness, but not in regions with unaffected BM organization, hemidesmosome morphology of mutants appeared compromised (Fig. 6C–F). Similarly, overall assessment of the MTJ by TEM revealed structural alterations characterized by disorganized ECM deposition at the muscle/tendon focal contact region, with wider, but less branched digitations of tendon material into the skeletal muscles of *Hmcn1* mutants (Fig. 6G, H; n = 5/5 mutants). The BM at the MTJ also appeared to be wider due to an increased thickness of the lamina densa, leading to an almost complete withdrawal of the lamina lucida (Fig. 6I–L), seen in 53.3 ± 8.3% of the investigated MTJ digitations of three mutants, compared to 8.7 ± 1.8% in three control siblings (p = 0.006). Also, in particular at the tips of the tendon interdigitations of *Hmcn1* mutants, regions directly underneath the BM of the MTJ were devoid of collagen fibers (Fig. 6I–L), resembling the phenotype formerly described for the skin blisters of zebrafish *hmcn1* mutants^13^. Together, this points to an essential role of mouse Hmcn1 for proper BM integrity. Compromised BM integrity in mutants in turn seems to affect the linkage of the BM to the overlying epithelial tissue, as reflected by the compromised hemidesmosome structure observed in the skin of mouse *Hmcn1* mutants, and to the underlying mesenchymal tissue, as reflected by the observed compromised connection of the BM to the tendinous collagen fibers in the MTJs of mouse *Hmcn1* mutants. These phenotypic traits are in line with the formerly described function of Hmcn for BM integrity and tissue linkage and/or separation in invertebrates.

## Discussion

Here we report Hmcn1 and Hmcn2 as new components of specialized cellular microenvironments in connective tissues. Based on newly raised specific Hmcn1 and Hmcn2 antibodies and in situ probes we determined their tissue distribution in embryonic and postnatal tissues and identified a new requirement of Hmcn1 for basement membrane integrity.

Genetic ablation of Hmcn1 in mice did not lead to embryonic lethality as previously reported^17^. As the underlying cause of this observed lethality, it was hypothesized that Hmcn1 aids cleavage furrow maturation and contractile ring formation extracellularly during cytokinesis^8^. Our analysis of Hmcn1 expression and localization during murine embryogenesis confirmed its localization at the cleavage furrow during the two-cell stage; however, genetic ablation of Hmcn1 by our conventional gene targeting approach did not affect embryonic and fetal development (Fig. 4B–J). This finding is in line with a recent report of a CRISPR/Cas9-induced *Hmcn1* loss-of-function allele. Similar to our findings, these *Hmcn1* mutants did not show abnormalities in any of the tissues and organs in which Hmcn1 was expressed and deposited^15^. As a possible explanation for this seeming discrepancy, it was speculated that different Hmcn1 isoforms might have been targeted in the different alleles^15^, and that early preimplantation development might require isoforms only affected by the initial^17^, but not by the more recently reported *Hmcn1* alleles (^15^ and this study). Yet, in the immunofluorescence analyses described in this study, we could detect Hmcn1 protein in 2–8 cell stage wild-type, but not in our Hmcn1 mutant embryos, using both N-terminal (Fig. 4C, D) and C-terminal (DW, JZ and MH, unpublished observation) polyclonal anti-Hmcn1 antibodies–which indicates that we did also target early Hmcn1 isoforms. Only the availability of a larger set of antibodies detecting the different domains of Hmcn1 protein and a complete set of mutant mouse lines deficient in all Hmcn1 isoforms may allow to clarify these discrepancies.

By generating additional antibodies against Hmcn2, we were also able to show that Hmcns display a mutually exclusive expression and distribution pattern in several embryonic and postnatal connective tissues. While Hmcn1 appears to be primarily expressed by resident cells of the mesenchyme, Hmcn2 seems to be produced by epithelial cells (Figs. 1, 2, 3). This suggests that Hmcns are not capable to functionally compensate for each other. Furthermore, the recent findings that, similar to *Hmcn1* single mutants, *Hmcn1*^−/−^; *Hmcn2*^−/−^ double mutant mice do not show any overt phenotype^15^ suggests that lack of Hmcns may be compensated by other ECM proteins, potentially by other members of the fibulin family. The extracellular deposition of Hmcn1 as supramolecular fibers associated with BMs is similar to the localization of members of the short fibulin family^18^. Short fibulins are found in elastic tissues in association with fibronectin fibers, fibrillin microfibrils and elastic fibers^1,19–22^. Therefore, it seems plausible that also Hmcns may require the fibronectin/fibrillin/elastic fiber scaffold for their proper ECM deposition. As our previous studies in zebrafish showed, only the mutual ablation of *hmcn2* and *fbln1* resulted in detrimental phenotypic consequences such as the formation of blisters in the trunk^14^. Thus, it remains to be determined which short fibulin may partner with which Hmcn to exert a functional activity in connective tissues. Further studies are required to address this question which is also relevant to elucidate the underlying pathological pathways of Fraser syndrome. Based on our previous studies in zebrafish, we hypothesized that *HMCN1* and *HMCN2* could be implicated in the pathogenesis of Fraser syndrome^13^. However, the absence of connective tissue defects in *Hmcn1*^−/−^ mice indicates that loss of mammalian Hmcn1 does not lead to Fraser syndrome-like connective tissue defects, possibly due to functional redundancies with other fibulins. Another possible reason for the Fraser syndrome-like phenotype of *hmcn1* mutant zebrafish, but not of *Hmcn1* mutant mice, could be differences in the genetic properties of the mutant alleles. The mutant mouse alleles are most likely amorphic (loss of Hmcn1 function), while the zebrafish alleles, characterized by single amino acid residue exchanges or C-terminal truncations, could elicit antimorphic or neomorphic effects, with mutated versions of Hmcn1 protein with aberrant activities/binding properties that cause dysregulated development and homeostasis of connective tissues. The generation of mutant *Hmcn1* and *Hmcn2* knock-in mouse models or the transgenic overexpression of mutant *Hmcn1* and *Hmcn2* variants may allow to test whether such dominant negative effects of mutant Hemicentins may phenocopy Fraser syndrome-like features in mice. Such experiments would also allow to investigate a potential impact of Hmcn1 or Hmcn2 deficiency on the network formation of short fibulins in tissues, which, when disrupted, were shown to result in deficient elastic fiber formation with severe connective tissue consequences such as syndactyly, contractures, cutis laxa, aortic aneurysm formation and dissection^18,23–25^.

Strikingly, however, our ultrastructural analysis also revealed a requirement of wild-type Hmcn1 per se for proper BM integrity at the DEJ and MTJ (Fig. 6), which had not been reported so far. The observation of an abnormal widening of the BM in the absence of Hmcn1 suggests that Hmcn1 structurally contributes to the BM architecture. Our observed co-localization with the BM marker laminin-α2 chain in skin is in line with this idea. However, it still remains unclear how Hmcn1 interacts with BM proteins. In addition to its potential association with the short fibulin network known to interact with BMs, it is conceivable that Hmcn1 makes direct contact with integral BM components such as nidogens or laminins. Our TEM findings indicate that these unknown functional Hmcn1 interactions are required for maintaining BM integrity. Similarly, it has also been reported that Hmcn2 is crucial for proper BM-enclosure of the satellite cell niche^12^. In addition, our findings together with recent reports confirmed a BM localization of Hmcn1 in kidneys^15^. This is in support of other reports mentioning Hmcn1 as a potential marker for renal pathophysiology^26^ and glomerular damage^27^. Future interaction studies of Hmcns and BM components will further elucidate a potential unknown function of Hmcns in BM organization. Of note, such interactions and functions of Hmcns might be evolutionary conserved throughout the animal kingdom. Thus, a similar requirement for Hmcn for BM integrity has been revealed via genetic studies in nematodes and flatworms^9–11^. In addition to BM integrity itself, the consequences of compromised BM integrity for tissue linkage or separation caused by the loss of *hmcn*/*Hmcn1* seem to be evolutionarily conserved as well. Thus, both in *C. elegans* and in zebrafish and mouse, their loss leads to compromised anchoring of juxtaposed BMs via the interjacent ECM, e.g. between the uterine and gonadal walls in *C. elegans*^9^, between the two adjacent epidermal BMs in the zebrafish body fin^13^, and, possibly, between adjacent BMs in the interdigitations of the MTJs in the mouse (this study, Fig. 6). In addition to such linkage to the underlying mesenchymal tissue, compromised BM integrity also appears to have implications to the linkage to the overlying epithelial tissue, indicated by compromised hemidesmosome architecture in the skin of both *C. elegans*^6,7^ and mouse (this study, Fig. 6) *hmcn*/*Hmcn1* mutants. Future studies have to reveal whether the requirement of Hmcn for tissue/stem cell separation and regeneration, as evident in planarians^11^, is conserved in vertebrates as well, for instance during cutaneous wound healing, when Hmcn1 and Hmcn2 are up-regulated in fibroblasts and basal keratinocytes at the wound edges of injured mouse (this study, Supplement to Fig. S2_2). Of note, mouse Hmcn1 has been implicated with fibrosis caused by ventricular cardiac fibroblasts^28^, suggesting that it might also contribute to pathological processes that occur during wound healing.

Despite such apparent structural changes of BMs found in DEJs and MTJs of mutant mice, *Hmcn1*^−/−^ mice performed normally in our biomechanical and locomotion tests. This could be due to the rather heterogeneous and mosaic occurrence of such BM alterations, with affected regions right next to unaffected regions both in the DEJs and the MTJs of *Hmcn1*^−/−^ mice. The reasons for this uneven expressivity of the BM defects and the mechanisms of the likely genetic compensation in unaffected regions are largely unclear. Complementation of the loss of Hmcn1 via Hmcn2 seems unlikely, as they are distributed in a complementary and mutually exclusive, rather than an overlapping manner. Thus, we found Hmcn2 to be localized in the epidermis and endomysium of muscle fibers, while Hmcn1 was found in dermis and tendon, thereby likely supporting the physical connection of both skin compartments as well as tendon integration with the muscle. Thus, rather than Hmcn2, other fibulins might compensate for the loss Hmcn1, similar to our previous findings for Hmcn2 and Fbln1 in zebrafish embryos, so that only the mutual loss of both may lead to significant functional biomechanical consequences. Therefore, a comprehensive co-localization analysis of Hmcns and short fibulins in the respective connective tissue compartments should be considered. In addition, functional challenging of skin and myotendinous junctions in *Hmcn1*^−/−^ single and in *Hmcn1*^−/−^*, Hmcn2*^−/−^ double mutants might yield informative results about the structural/biomechanical functions Hemicentins exert in connective tissues that are not apparent under unchallenged conditions.

In summary, our findings highlight Hemicentins as network-forming connective tissue components, which facilitate contact points of the ECM architecture with BMs and the cell surface in specialized connective tissue microenvironments. Our findings provide evidence for a complementary localization pattern of Hemicentins in murine tissues where Hmcn1 is detected in mesenchymal tissues while Hmcn2 is expressed by epithelial layers. *Hmcn1* ablation in mice did not yield significant anatomical, biomechanical or locomotion phenotypes, suggesting that it may partner with other ECM components to exert proper tissue functions. Currently, the contribution of Hemicentins to human pathology is not clear. *HMCN1* mutations have been reported to correlate with age-related macular degeneration. However, the underlying mechanisms have not been elucidated so far. Future studies are also required to elucidate the potential contribution of Hemicentins to the pathology of Fraser syndrome and connective tissue disorders with overlapping features.

## Materials and methods

All methods were carried out in accordance with the ARRIVE guidelines and all other relevant guidelines and regulations.

### Animals

Mouse handling was performed under standardized specific pathogen-free conditions. The mouse handling experimental protocols were examined and approved by the Agency for Environment and Consumer Protection of the City of Cologne (576.1.36.6.G13/15 Be) and by the State Agency for Nature, Environment and Consumer Protection North Rhine Westphalia (LANUV NRW; 84-02.05.40.17.014, 84-02.04.2015.A034). C57BL/6 and CB20 mice were ordered from Charles River, Taconic or Jackson Laboratories. Flp deleter mice^29^ were kindly provided by the animal facility at the Max Planck Institute for Metabolism Research, Cologne, Germany.

### Generation of *Hmcn1*^−/−^ mice

Targeting vectors were engineered via bacterial artificial chromosome (BAC) recombination obtained from BAC PAC Resources Center (Children’s Hospital Oakland Research Institute), *Mus musculus* C57BL/6 J male BAC library. The clone RP24-236G13, containing large sequences of *Hmcn1*, was used to engineer the long recombination arms of the targeting vector p*Hmcn1*-exon1 in two steps. The first part included the amplification of the neomycin resistance cassette containing gb2 and PGK promoters flanked by FRT sequences from pCTAP vector using the primers: 5Hmcn1-5′ CAG TAT TCC TGG TGG CTC TTT TTC GTT CTT CCC TAG CTG GAG ATG GGA CTG CCT TAA GCT TGG AAA AGC TGG CGC and 3Hmcn1-5′ AAA TAA TAG AAT TAT GAA TCA CCA AGC ACA ACA AATACA GAG AGT AAG TAT CGA TCG CCT AGG GGT AAC CG 3′. The second step was the amplification of a smaller vector, pACYC177, that was amplified using the primers: 5Hmcn1ev-5′ GAG ACT AGA TAG CCT ATG GAA GGG GGA CTT AGA GGA AGT GAG AAG AGT TAT CTT AGA CGT CAG GTG GCA CT 3′ and 3Hmcn1ev-5′ GGA CTG AAG ACT TGG TTG GAC TGT GAG GAA GGA ACA ACA GGA GAG CCA GCA CCG GTG CGT CAG CAG AAT AT 3′ for recombination with the BAC containing Hmcn1 homology arms flanking the selection marker. The linearized targeting vector was electroporated into Bruce4 embryonic stem cells. Neomycin-resistant clones were screened by PCR and Southern blot for homologous recombination and some positive clones were injected into CB20 blastocysts by the technical team of the transgenesis facility at the Institute of Genetics, Cologne. After identification of germline transmission, two independent lines deriving from different ES cell clones were established, and mice were outcrossed at least ten times in C57BL/6 J background. *Hmcn1*^+*/*+^ control mice (H1^con^) refer to wild-type littermate controls obtained from mating of heterozygous *Hmcn1*^+*/*−^ parents. Identical results were obtained for mice from both lines.

### Histology, in situ hybridization and immunofluorescence

All tissue or whole-mount embryos were either fixed for 4 h in 4% paraformaldehyde before paraffin embedding (in situ hybridisation, histology), or frozen unfixed in optimal cutting temperature compound (OCT, Sakura, Torrance, CA) (Immunohistochemistry). From paraffin embedded and cryopreserved samples 6 µm and 12 µm thick sections were generated.

For histology, the sections from paraffin embedded samples were stained with Hematoxilin-Eosin (Merck), Giemsa (Merck) or Masson–Goldner Trichrome staining (Merck) following the manufacturer’s protocol.

For in situ hybridization, *Hmcn1* and *Hmcn2* probes were amplified by PCR from mouse cDNA spanning several introns. The *Hmcn1* probe was designed in a region covering mostly 3′ UTR (1.4 kb, exon 107–108) and the *Hmcn2* probe covered the G2 domain and EGF 1–4 (1.3 kb, exon 81–96). Sections from paraffin embedded mouse embryos were processed as previously described ^30^. Briefly, the pre-treatment of the sections consisted only of proteolytic digestion for 5–15 min at 37 °C with 20 mg/ml proteinase K and then re-fixed for 20 min in 4% formaldehyde/0.2% glutaraldehyde. Sections were hybridized overnight at 70 °C in hybridization buffer (50% formamide/SSC, 1% blocking solution (Roche), 5 mM EDTA, 0.1%Tween-20, 0.1% Chaps (Sigma; St. Louis, MO), 0.1 mg/ml heparin (Becton-Dickinson; Mountain View, CA), and 1 mg/ml yeast total RNA (Roche)). After hybridization, sections were rinsed in 2 × SSC, pH 4.5, washed three times for 30 min at 65 °C in 50% formamide/2 × SSC, pH 4.5, followed by three 5-min washes in PBST. Probe bound to the section was detected using sheep anti-digoxygenin Fab fragment covalently coupled to alkaline phosphatase, and NBT/BCIP as chromogenic substrate, essentially according to the manufacturer’s protocol (Roche). Sections were washed with double-distilled water, dehydrated in a graded ethanol series and xylene, and embedded in Entellan.

For immunofluorescence, cryosections were fixed for 10 min with 4% paraformaldehyde, followed by several washes with 0.1% Triton X-100 in PBS. After washing, the sections were blocked with 10% sheep serum for 1 h and then incubated with the required primary antibodies in blocking solution. Polyclonal antibodies against an N-terminal fragment of mouse Hemicentin-1 (Hmcn1, 1653–2275aa) and against a C-terminal fragment of mouse Hemicentin-2 (Hmcn2, 4429–4753aa) were generated in our laboratory^31^. Monoclonal antibodies for this study were used as follows: anti-TenascinC (T3413, 1:200, Sigma), anti-SMA (A2547, 1:1000, Sigma), anti-Laminin-α2 chain (ALX-804-190; 1:100, Enzo). After overnight incubation at 4 °C the sections were again washed with PBS and incubated with the appropriate secondary antibodies conjugated to Alexa 488 or 555 (Molecular Probes, Eugene, OR, USA) at RT for 2 h. For labelling F-actin, embryos were incubated with Phalloidin-TRITC (Sigma-Aldrich), and DAPI (4′,6-diamidino-2-phenylindole, dihydropchloride) was used to counterstain the nuclei. Confocal images were obtained using a Zeiss LSM 710 or LSM 700 confocal microscope, 40 × /1.1 W Korr LD C-Apochromat, or 20 × /0.8 Plan-Apochromat objective and Zen 2.3 SP1 software. Images were processed using Fiji/ImageJ software to obtain maximum intensity projections, and for adjustment of brightness and contrast.

### Dermis thickness measurement

Skin samples were collected from dorsal mouse skin of newly born (P1), 4-weeks old (P28) and 1-year old (1Y) mice. Skin thickness was quantified blinded to the genotype of the mice. Multiple regions across a single long biopsy of skin were examined and measured at three distinct sites and averaged to determine the dermal thickness for each mouse.

### Wounding and preparation of wound tissues

Preparation of wound tissue for immunohistochemistry was performed as described previously^32^. Briefly, mice were anesthetized, shaved, and two 6-mm diameter full-thickness wounds were generated using a standard biopsy puncher (Stiefel, Offenbach, Germany). For histological analysis, wounds were excised at 4 days post injury (dpi). Wounds were disected in the caudocranial direction, and the tissue was embedded in OCT compound (Tissue Tek, Miles, Elkhart, IN, USA).

### Transmission electron microscopy

The samples of back skin and myotendinous junction (Achilles tendon/soleus) from 1-year old mice were isolated and fixed in buffer (2% paraformaldehyde, 2% glutaraldehyde, and 0.1 mol/L cacodylate buffer at pH 7.35) for 24 h. After the tissue was processed according to a standard protocol and embedded in Epon-Araldite. The ultrathin Sects. (50–100 nm) were analysed via an EM109 electron microscope (Zeiss, Oberkochen, Germany).

### Western blot

Adult skin lysates were prepared by snap freezing in liquid nitrogen and homogenized by pestle and mortar. The homogenate was put in extraction buffer (150 mM NaCl, 50 mM Tris (pH 7.4), 2 mM EDTA, 1% Nonidet P-40, Protease inhibitor cocktail (Roche)) and further homogenized with a rotor–stator homogenizer. Equal amounts of proteins (100 µg) were resolved in a 6% polyacrylamide gel and wet-transferred to a nitrocellulose membrane overnight at 4 °C in transfer buffer (25 mM Tris–Cl, 192 mM Glycin, 20% Methanol). After blocking buffer (4% BSA; 0.1% Tween-20, 1 × TBS), primary antibody anti-Hmcn1 (1653-2275aa, 1:10,000, self-made) or control anti-Vinculin (EPR8185, 1:6000, Abcam) were applied.

### Collection of mouse embryos (E1.5)

All mice used for mating experiments were between 8 and 14 weeks of age and were maintained in a 12 h-light and 12 h-dark cycle. Females were checked for vaginal plugs in the morning, which was set as embryonic day 0.5 (E 0.5). The next day (E1.5, 2-cell stage), pregnant females were euthanized by cervical dislocation and the uterus was removed. Embryos were collected from oviducts by flushing twice with M2 medium and directly fixed with 4% paraformaldehyde for 10 min. Afterwards immunocytochemistry was performed as described previously^17^. Briefly, the embryos were washed with neutralization solution (50 mM NH_4_Cl in PBS) for 15 min and permeabilized in PBS containing 0.25% Triton X-100 for 10 min. The samples were blocked with 5% BSA for 1 h and subsequently incubated with primary antibodies overnight at 4 °C followed by incubating with appropriate secondary antibodies diluted according to manufacturer’s recommendation for 1–2 h at room temperature.

### Locomotion tests

#### Rotarod test

Mice were tested on a turning, corrugated rod (Jones & Roberts, TSE systems, Bad Homburg, Germany) three times (trials) for two consecutive days. Wild-type control (n = 8) and *Hmcn1*^−/−^ (n = 7) male mice of age 12 weeks were tested. On day 1, mice were first acclimatized to the rotarod at slow, constant speed (4 rpm) for a maximum duration of 3 min. Then three trials were performed with the accelerating rod, starting with 5 rpm up to 40 rpm within 5 min. On the following day, again the three trials with the same accelerating rod were carried out. The performance of the mice was evaluated by scoring the latency to fall down.

#### Treadmill

The running groups were trained on a treadmill (Exer -3/6, Columbus Instruments International, Columbus, OH, USA) at a velocity of 20 m/min for 30 min per day, 5 days per week, over a total intervention period of 6 weeks. The training was divided into a morning (7:00–8:00 h) and a late afternoon (5:00–6:00 h) session, 15 min each. The LEVEL group was trained on level, whereas the DOWN group was trained on a 20° decline. During the first week, the running groups were accustomed to the treadmill running by a daily increase of the velocity and the declination, respectively. Mice were exercised on a treadmill for three consecutive days (Exer -3/6, Columbus Instruments International, Columbus, OH, USA). Wild-type control (n = 9) and *Hmcn1*^−/−^ knockout (n = 9) male mice of age 14 months were tested. On the first day of the experiment, the mice were subjected to 27 min of running at a speed increasing from 8 to 24 m/min. The second day the mice rested and during the third day, the duration of running was increased to 40 min but with a constant speed of 20 m/min and an incline of + 5°.

#### Gait (footprint) analysis

Blue and red nontoxic drawing ink was applied by using a cotton bud to the hind and fore paws, respectively. A new sheet of graph paper was placed on the floor of the runway for each test run. 14 months old male mice were split into untrained group (wild-type control, n = 9 and *Hmcn1*^−/−^, n = 9) and trained (wild-type control, n = 6 and *Hmcn1*^−/−^, n = 6) group. The trained mice were trained daily for 5 days 30 min on a treadmill with a constant speed of 12 m/min at a 10° incline. The resulting footprint tracings were analyzed, measuring four parameters: (i) stride length for hind paws, determined by measuring the distance between each paw print on the right side of the body; (ii) hind and front base width by measuring the distance between the right and left hind paws (hind base) and the right and left front paws (front base); (iii) overlap between fore and hind footprints. Gait parameters were determined by drawing parallel lines through the center of each footprint and measuring distance (in mm) between the appropriate lines.

#### Limb clasping assay

The mice were suspended by the base of the tail and their behaviors were recorded. Wild-type control (n = 9) and *Hmcn1*^−/−^ knockout (n = 9) male mice of 6 and 14 months of age were tested. Tail suspensions were performed ten times and stopped at the first clear incidence of hindlimb clasping. Three separate trials were taken over the course of the test, and the average number of three trials were used for statistical analysis.

### Biomechanical analysis

The tensile strength of back skin and the Achilles tendon was analysed using a material testing machine (model Z2.5/TN1S, Zwick, Ulm, Germany) with a 100 N (skin) or 10 N (myotendinous junction) load cell recording force–deformation curves. Two hourglass-shaped strips of back skin (25 mm long, 5 mm in the middle, 10 mm width at the ends), and the Achilles tendons together with m. gastrocnemius, m. soleus and calcaneus were harvested, wrapped in saline‐soaked gauze and stored at − 20 °C until mechanical testing. We tested control (n = 5) and Hemicentin1 knockout (n = 3) 16 months old male mice. After preloading (skin: 0.05 N, 0.1 mm/s; myotendinous junction: 0.01 N, 10 mm/min), specimens were loaded until failure (skin: 15 mm/min; myotendinous junction: 10 mm/min). The ultimate load (N), deformation (mm) and energy (mJ) were determined from the load-deformation curve. The stiffness (N/mm) was calculated from the slope of the linear part of the force–elongation curve.

### Micro‐CT analysis

Microstructural architecture of the femur from 15 months old *Hmcn1*^−/−^ (n = 7) and control mice (n = 9) was analyzed using a high-resolution µCT scanner (μCT 35, Scanco Medical AG). Isolated bones were scanned with an isotropic voxel-size of 7 × 7 × 7 μm using 70 kVp energy, 114 μA current and 400 ms integration time. To remove image noise, gray-scale data of raw images were preprocessed using a 3D Gaussian filter algorithm. The mineralized tissue was separated from soft tissues by a global thresholding procedure^33^. The segmentation steps were applied with support = 1.0, sigma = 0.8. The image data were segmented using different thresholds (27% for trabecular and 29% for cortical bone) of the maximum gray scale values.

### Statistical analysis

Statistics was performed using PRISM (Graph Pad Software) and SPSS software. Statistical significances of differences were analyzed with two-tailed, unpaired Student’s *t* tests, and, in case of the TEM BM data (Fig. 6), additional nonparametric Mann–Whitney-*U*-tests. All data are presented as mean ± standard error of the mean (SEM), and a *p *value < 0.05 was considered significant. The results are presented as the average of at least three independent experiments unless otherwise stated in the legends.

## Supplementary Information

Supplementary Figures.
