# Supplementary material for: Hemicentin-1 is an essential extracellular matrix component of the dermal–epidermal and myotendinous junctions
Source: Sci Rep. 2021 Sep 9;11:17926. doi: 10.1038/s41598-021-96824-4 (PMC8429575; doi:10.1038/s41598-021-96824-4)
Supplement: Supplementary file 1 — Supplementary Figures. [file 41598_2021_96824_MOESM1_ESM.pdf]

Supplemental Information

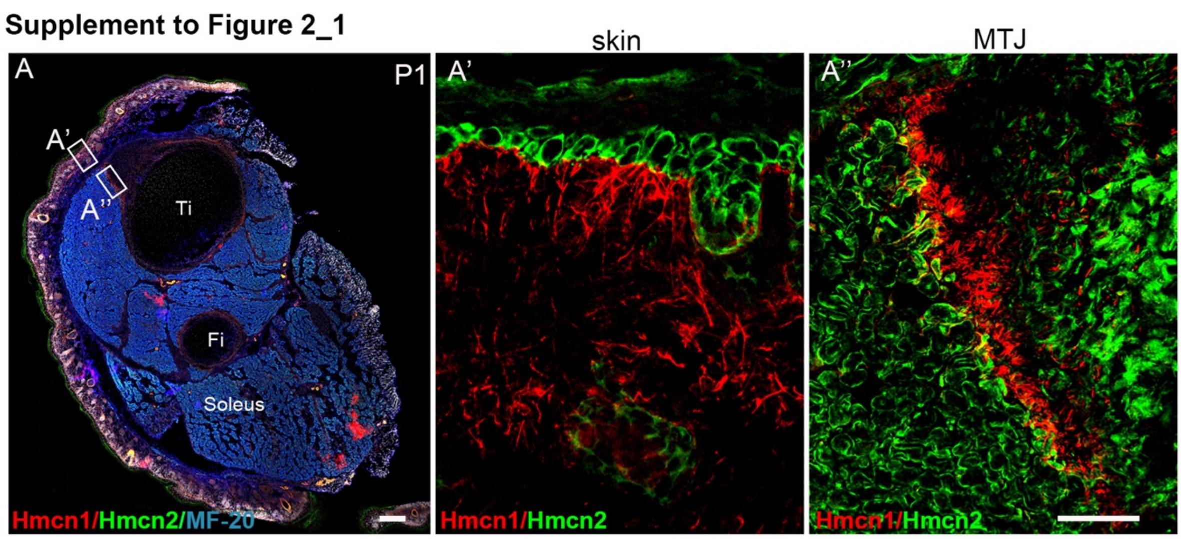

Supplementary Figure S2_1. Differential localization of mouse Hmcn1 and Hmcn2 at the dermal-epidermal junction (DEJ) and muscle-tendon junction (MTJ).

(A) Representative cross section through calf of neonate, immuno-stained with antibodies against Hmcn1 (red), Hmcn2 (green), MF-20 (blue). Higher magnification images of DEJ **(A´)** and MTJ **(A´´)** show clear differential expression of Hmcn2 in epithelial (epidermis, skeletal muscle)) and Hmcn1 in mesenchymal (dermis, tendon) tissue. Scale bars A= 200 µm; A´, A´´= 50 µm.

Supplement to Figure 2_2

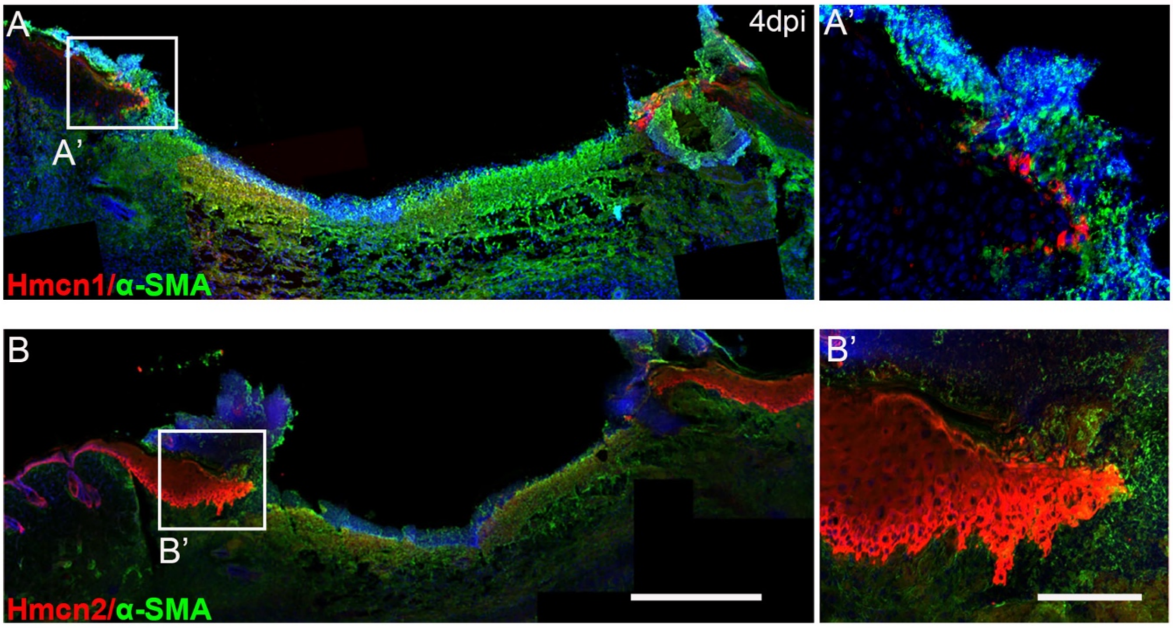

Supplementary Figure S2_2. Hmcn1 and Hmcn2 protein localization during cutaneous wound healing.

(A) Hmcn1 (red) is up-regulated in the dermis adjacent to the epidermal tongues of closing wounds. **(B)** Hmcn2 (red) is strongly up-regulated in the basal keratinocytes within the epidermal tongues of closing wound. α-SMA (green), marker for myofibroblast differentiation and DAPI (blue) counterstains nuclei. Scale bars: A, B= 500 µm; A´, B´= 100 µm.

Supplement to Figure 3_1

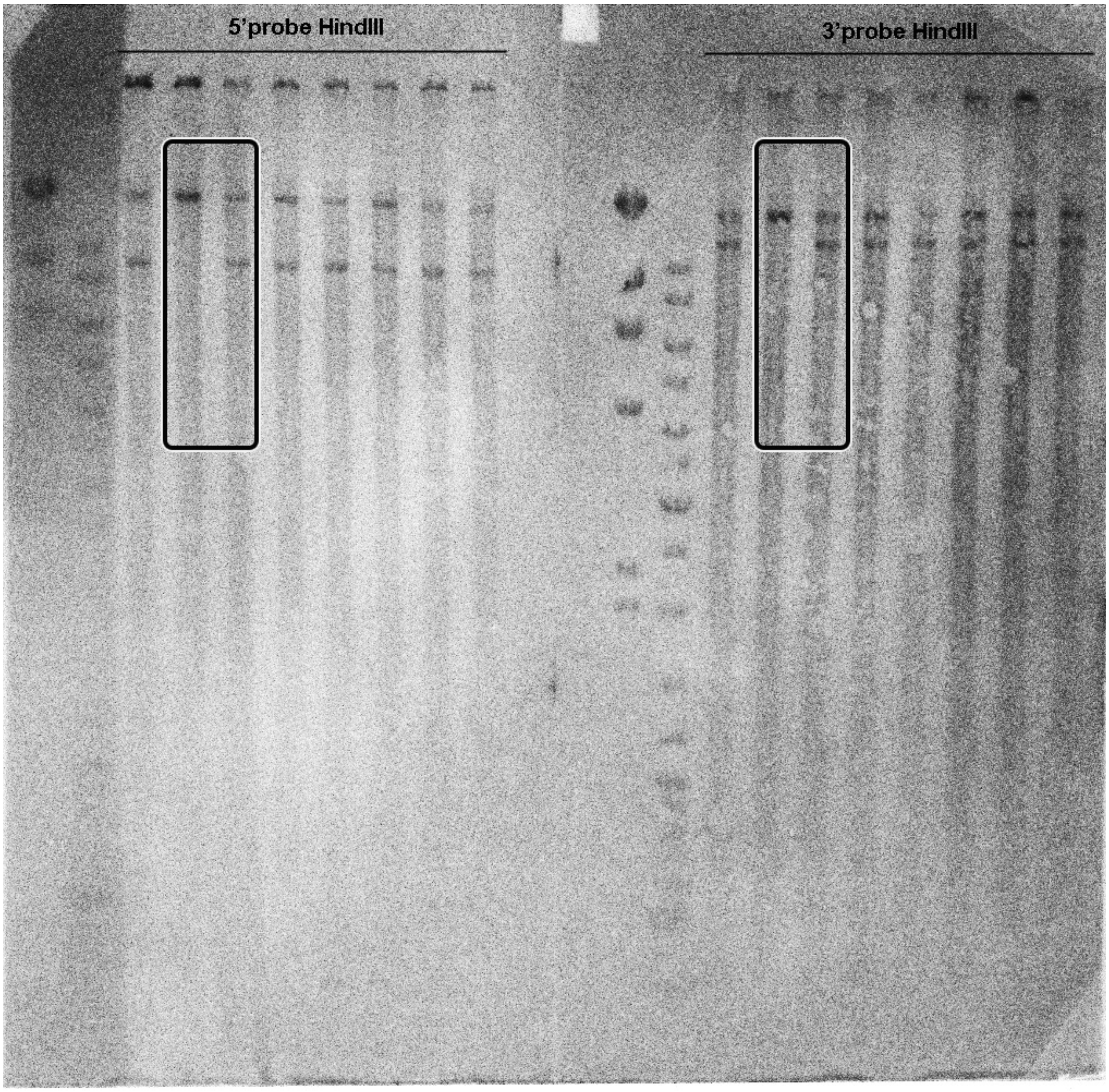

Full-size Southern blots of *HindIII*-digested genomic DNA from ES cells using 5´and 3´ *Hmcn1* probes. The regions shown in Fig. 3b are boxed.

Supplement to Figure 3_2

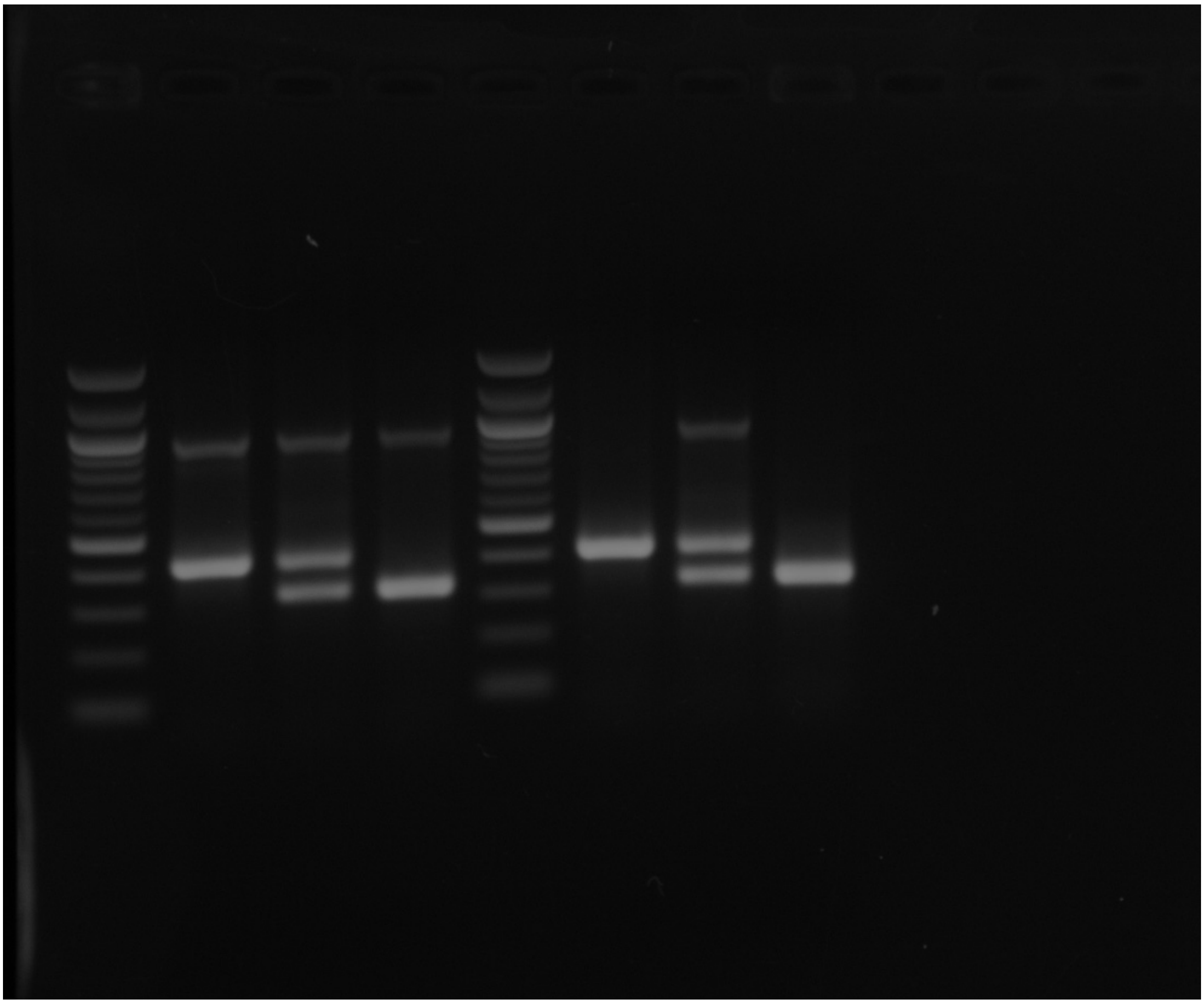

Full-size and unprocessed image of ethidium bromide gel of PCR products for genotyping of *Hmcn1* mutant mice, parts of which are shown in reverse black and white as Fig. 3C.

Supplement to Figure 3_3

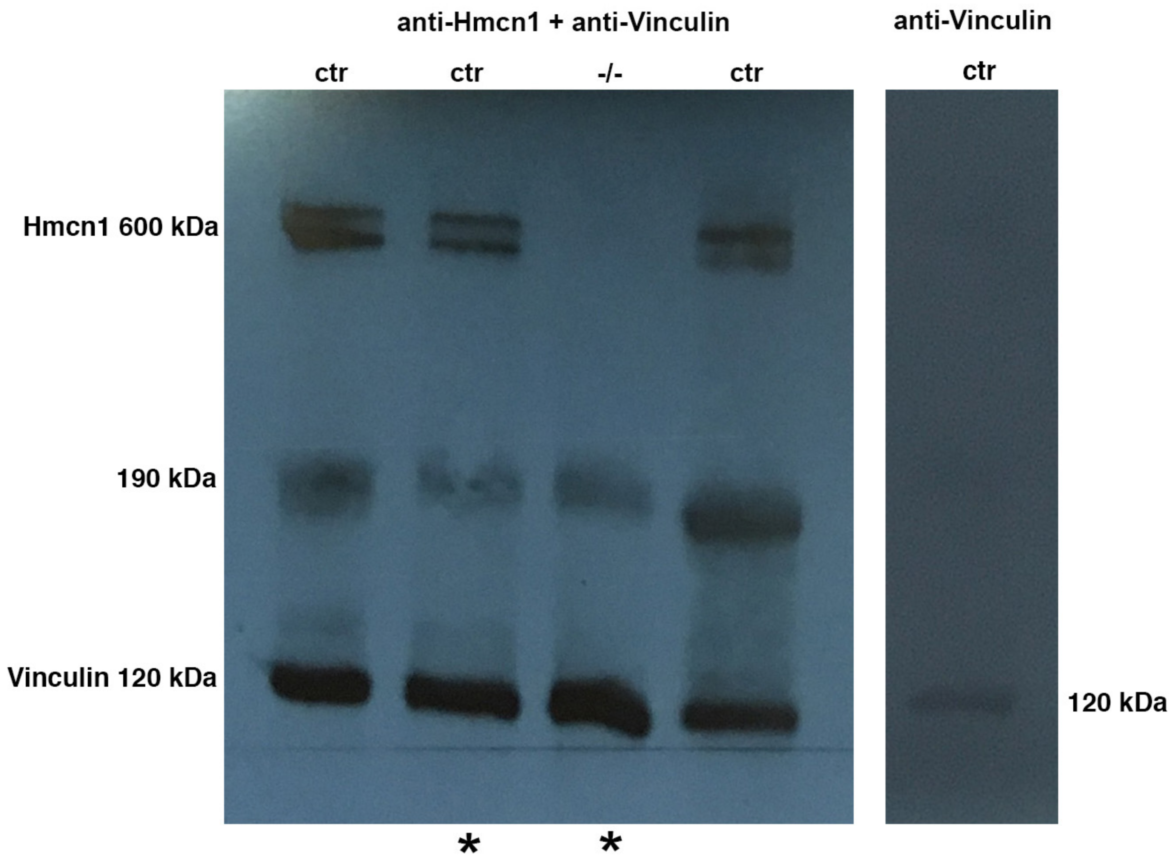

Western blot of protein extracts from *Hmcn1* mutant (-/-) and control siblings (crt) probed with anti-Hmcn1 and anti-Vinculin antibodies (left panel). The 120 kDa Vinculin band was absent in blots solely probed with the anti-Hmcn1 antibody (not shown), but present in blots solely probed with the anti-Vinculin antibody (right panel). The lanes shown in Fig. 3D are marked by asterisks.

Supplement to Figure 5_1

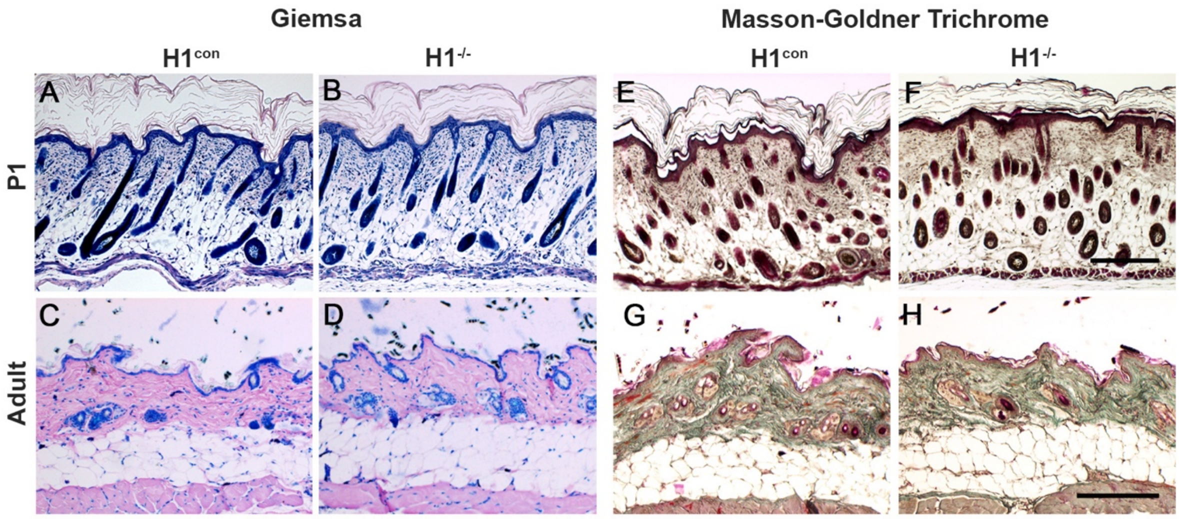

Supplementary Figure S5_1. Histological analysis of the back skin in newborn and adult mice. **(A-D)** Giemsa and **(E-H)** Masson-Goldner Trichrome staining show comparable dermal/ epidermal morphology, including skin appendages and collagen content between H1^{con} and H1$^{-/-}$ mice. Scale bar represents 200 µm. Abbreviations: H1$^{-/-}$, *Hmcn1*$^{-/-}$ mutants; H1^{con}, sibling controls of *Hmcn1*$^{-/-}$ mutants.

Supplement to Figure 5_2.

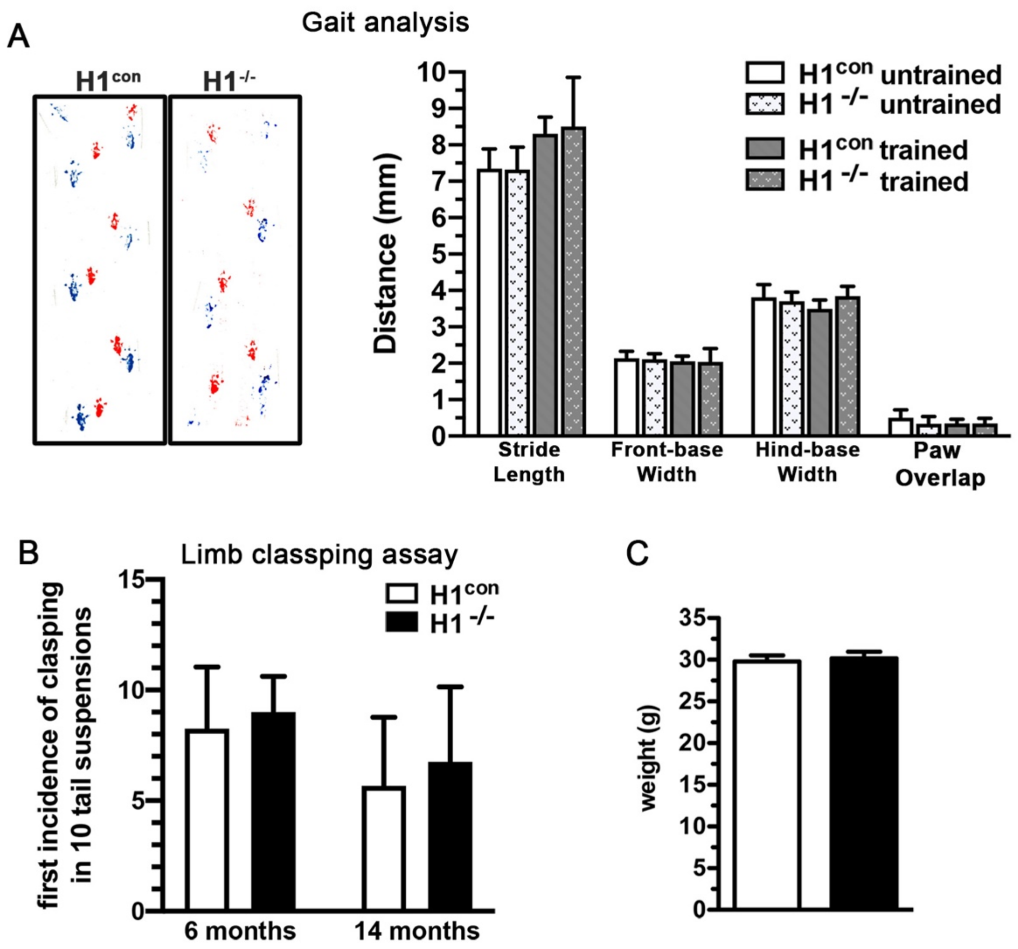

Supplementary Figure S5_2. Locomotion tests

(A) Footprint gait analysis of H1^{con} and H1$^{-/-}$ mice that were trained or untrained. Non-toxic paint was applied to record the footprint placement pattern. Stride length, stride width, hind-base width and paw overlap were measured as specified in Materials and Methods, revealing no significant differences between 14-months old control and H1$^{-/-}$ mice. (**B**) Hindlimb clasping assay was tested in control (n=9) and H1$^{-/-}$ (n=9) mice of age of 6- and 14-months. Tail suspensions were performed up to ten times and stopped at first clear incidence of hindlimb clasping, revealing no significant differences between control and H1$^{-/-}$ mice. (**C**) No significant difference in body weights of tested 14-months old control and H1$^{-/-}$ mice. Abbreviations: H1$^{-/-}$, *Hmcn1*$^{-/-}$ mutants; H1^{con}, sibling controls of *Hmcn1*$^{-/-}$ mutants.

Supplement to Figure 5_3

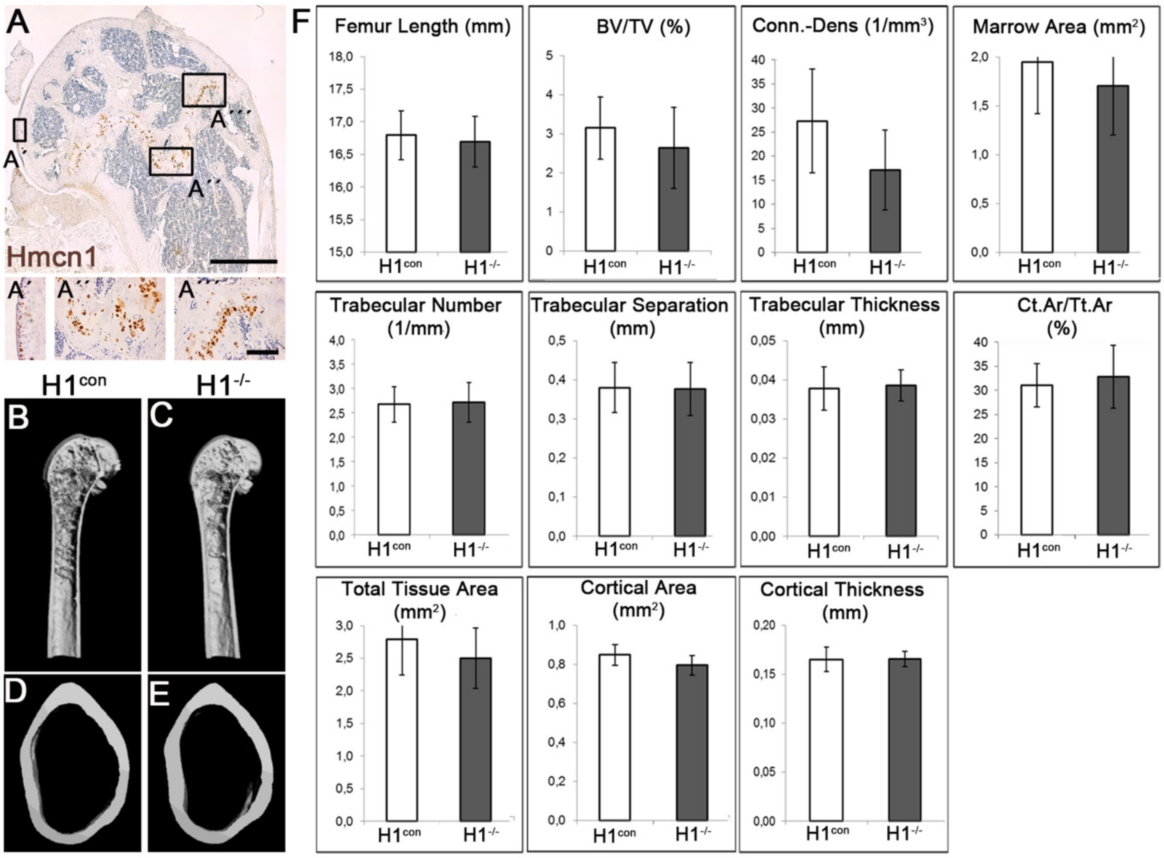

Supplementary Figure S5_3. µCT analysis of trabecular and cortical area of the femur

(**A**) Immunohistological staining against mouse Hmcn1 (brown) shows the localization in the articular cartilage (**A´**) and in the hypertrophic chondrocytes of the growth plate (**A´´, A´´).** Nuclei were counterstained with hematoxylin**. (B-E)** Representative µCT 3D images of the femur of 15 months-old mice revealed no difference in bone structure between H1^{con} controls and H1$^{-/-}$ mutants. Images show sagittal view of femur (**B, C)** or top view of the cortical bone of femur; 0.75mm around middiaphysis (**D, E**). (**F**) Quantification of the femur length, BV/TV (bone volume fraction), Conn.-Dens (connectivity density), marrow area, trabecular (Tt.Ar) and cortical area (Ct.Ar) of H1$^{-/-}$ mice (n=7) and H1^{con} control siblings (n=9), as shown in (B-E). Scale bars A=500 µm; A´, A´´, A´´´=25 µm. Abbreviations: H1$^{-/-}$, *Hmcn1*$^{-/-}$ mutants; H1^{con}, sibling controls of *Hmcn1*$^{-/-}$ mutants.
